# Association between the Mediterranean lifestyle, metabolic syndrome and mortality: a whole-country cohort in Spain

**DOI:** 10.1186/s12933-020-01195-1

**Published:** 2021-01-05

**Authors:** Mercedes Sotos-Prieto, Rosario Ortolá, Miguel Ruiz-Canela, Esther Garcia-Esquinas, David Martínez-Gómez, Esther Lopez-Garcia, Miguel Ángel Martínez-González, Fernando Rodriguez-Artalejo

**Affiliations:** 1 Department of Preventive Medicine and Public Health, School of Medicine, Universidad Autónoma de Madrid, IdiPaz (Instituto de Investigación Sanitaria Hospital Universitario La Paz), and CIBERESP (CIBER of Epidemiology and Public Health), Madrid, Spain; 2 Department of Environmental Health, Harvard T.H. Chan School of Public Health, Boston, MA USA; 3 Department of Preventive Medicine and Public Health, University of Navarra, IdiSNA, Pamplona, Spain; 4 Biomedical Research Network Centre for Pathophysiology of Obesity and Nutrition (CIBEROBN), Carlos III Health Institute, Madrid, Spain; 5 Department of Nutrition, Harvard T.H. Chan School of Public Health, Boston, MA USA; 6 IMDEA-Food Institute, CEI UAM+CSIC, Madrid, Spain

**Keywords:** Mediterranean lifestyle, Cardiovascular risk factors, Metabolic syndrome mortality, Cohort

## Abstract

**Background:**

Evidence is limited about the joint health effects of the Mediterranean lifestyle on cardiometabolic health and mortality. The aim of this study was to evaluate the association of the Mediterranean lifestyle with the frequency of the metabolic syndrome (MS) and the risk of all-cause and cardiovascular mortality in Spain.

**Methods:**

Data were taken from ENRICA study, a prospective cohort of 11,090 individuals aged 18+ years, representative of the population of Spain, who were free of cardiovascular disease (CVD) and diabetes at 2008–2010 and were followed-up to 2017. The Mediterranean lifestyle was assessed at baseline with the 27-item MEDLIFE index (with higher score representing better adherence).

**Results:**

Compared to participants in the lowest quartile of MEDLIFE, those in the highest quartile had a multivariable-adjusted odds ratio 0.73 (95% confidence interval (CI) 0.5, 0.93) for MS, 0.63. (0.51, 0.80) for abdominal obesity, and 0.76 (0.63, 0.90) for low HDL-cholesterol. Similarly, a higher MELDIFE score was associated with lower HOMA-IR and highly-sensitivity C-reactive protein (P-trend < 0.001). During a mean follow-up of 8.7 years, 330 total deaths (74 CVD deaths) were ascertained. When comparing those in highest vs. lowest quartile of MEDLIFE, the multivariable-adjusted hazard ratio (95% CI) was 0.58 (0.37, 0.90) for total mortality and 0.33 (0.11, 1.02) for cardiovascular mortality.

**Conclusions:**

The Mediterranean lifestyle was associated with lower frequency of MS and reduced all-cause mortality in Spain. Future studies should determine if this also applies to other Mediterranean countries, and also improve cardiovascular health outside the Mediterranean basin.

## Background

The Mediterranean diet (MedDiet) is the culturally rooted diet traditionally consumed by the populations living in the Mediterranean basin [1]. Despite some between-country variations, the MedDiet in Spain reflects a food consumption pattern rich in fruits and vegetables, legumes, whole grains, olive oil, fish and nuts, with greater consumption of white or lean meats than of red or processed meats, moderate consumption of dairy products, and intake of small amounts of wine, mainly red wine and with meals. In addition, the MedDiet reflects other influences of the Mediterranean culture, including a rich social interaction, “siesta” (short nap after meals), eating with friends and family, traditional recipes and culinary practices, and a substantial amount of physical activity; all these elements characterize the Mediterranean lifestyle, as represented in the MedDiet pyramid elaborated though a consensus of nutrition experts and the MedDiet Foundation [2].

There is robust evidence, accumulated over the last decades, of the beneficial effects of the MedDiet on chronic diseases, including the metabolic syndrome (MS) [3–5], type 2 diabetes [6–9] and obesity [10, 11] as well as cardiovascular disease [12–16], cancer [17] and all-cause mortality [13, 18–20], both in Mediterranean and non-Mediterranean countries.

More recently, epidemiological studies have started to evaluate the joint health effect of multiple health behaviors and biological CVD risk factors combined into a single predefined score [21–27]. For example, Li et al., found that adopting four out of five low risk lifestyle factors (never smoking, healthy body mass index (BMI), moderate-to-vigorous physical activity, moderate alcohol intake, and a higher diet quality) was associated with about a 10-year increase in life expectancy [25]. However, there is a lack of evidence about the joint and synergistic health effects of the different components of the Mediterranean lifestyle. The Mediterranean lifestyle (MEDLIFE) index is a score of the set of behaviors that best characterize the adoption of a traditional Mediterranean lifestyle [28, 29]. It was developed and validated in a Spanish working population [28, 29], and a higher score has been associated with lower frequency of CVD risk factors in 366 workers from Croatia [30], and reduced mortality in a Spanish cohort of university graduates. However, no previous research has evaluated the influence of the MEDLIFE index on cardiovascular health in the adult population of a whole Mediterranean country. Therefore, the aim of this study was to evaluate the association of the MEDLIFE index with the prevalence of the MS and other biological CVD risk factors, and with the risk of all-cause and CVD death, on a representative sample of the adult population of Spain.

## Research design and methods

### Study population

The Study on Nutrition and Cardiovascular Risk in Spain (ENRICA) is a prospective cohort study that consists of 13,105 individuals aged 18 years or older, who were recruited between June 2008 and October 2010 through random stratified cluster sampling to ensure that they were a representative sample of the non-institutionalized adult population of Spain. First, the sample was stratified by province and size of municipality. Second, clusters were selected randomly in two stages: municipalities and census sections. Finally, the households within each section were selected proportionally to the sex and age distribution of the Spanish population. Detailed information about the methods and sample collection process have been published elsewhere [31]. Trained and certified staff collected information in three stages: a phone interview and two subsequent home visits. The phone interview obtained data on sociodemographic factors, health behaviors, self-rated health and the existence of a previous medical diagnosis of a set of chronic conditions. In the first home visit, blood and urine samples were collected and sent to a central laboratory for analytical determinations; and in the second visit, a validated electronic diet history was obtained and a physical examination was performed [32]. Study participants have been followed-up to December of 2017 (mean follow-up to 8.7 years, SD 0.87) to assess their vital status and the cause of death.

Of the 13,105 participants at baseline, 921 were excluded because of missing information on diet or implausible reported energy intake (< 800 kcal or > 5000 kcal for men, and < 500 kcal or > 4000 kcal for women). We also excluded those lacking data on their clinical CVD risk factors (n = 115) and those who had a previous medical diagnosis of CVD (heart failure, myocardial infarction or stroke) (n = 262) or diabetes at baseline (n = 716). That left a sample size of 11,091 for the analyses (Additional file 1: Figure S1).

Study participants gave written informed consent, and the study was approved by the Clinical Research Ethics Committee of La Paz University Hospital in Madrid (Trial registration: NCT02804672).

### Study variables

#### Mediterranean lifestyle index

Baseline information on habitual food consumption in the preceding year was obtained with a validated computerized face-to-face diet history (DH-ENRICA), developed from that used in the European Prospective Investigation into Cancer and Nutrition (EPIC)-cohort study in Spain [32]. The diet history included 880 different foods and a set of 127 photographs that helped to quantify food portions. Standard food composition tables were used to estimate nutrient and energy intake [32].

Physical activity was ascertained using the validated European Prospective EPIC-cohort questionnaire [33]. Leisure-time activity was reported as the time spent in walking, cycling and other types of exercise, as well as in household chores, gardening and do-it-yourself activities. Time spent in sedentary behaviors, including while watching TV, at the computer use, reading, commuting, and listening to music was also obtained using the Nurses’ Health Study questionnaire validated in Spain [34]. Data regarding sleep, nap, conviviality and habits around meals were also self-reported.

Based on the above information, the MEDLIFE index was calculated following the criteria published by Sotos-Prieto et al. [28, 29]. Briefly, the MEDLIFE consists of 28 items divided into three blocks: (1) food consumption (15 items); (2) dietary habits (7 items); and (3) physical activity, rest and conviviality (6 items). However, we could not compute the item on water consumption for not having appropriate information in the ENRICA study. Each item of the score weights 0 (negative) or 1 point (positive), and the score ranged from 0 to 27 (best adherence) (Additional file 1: Table S1).

#### Outcome measurements

##### Clinical CVD risk factors

According to the harmonized definition [35], MS was defined as having at least three of the following five criteria: abdominal obesity (waist circumference ≥ 102 cm in men, or ≥ 88 cm in women); elevated blood glucose (≥ 100 mg/dl or treatment with antidiabetic drugs); high blood pressure (systolic ≥ 130 mmHg or diastolic ≥ 85 mmHg, or receiving antihypertensive drugs); triglycerides ≥ 150 mg/dl; and serum high-density lipoprotein (HDL) cholesterol < 40 mg/dl in men or < 50 mg/dl in women. Waist circumference was measured with a flexible non-stretchable tape at the midpoint between the last rib and the iliac crest after a normal expiration. Blood glucose was determined by the glucose oxidase technique after 12 h fasting. Blood pressure was measured by trained personnel using a standard protocol, with validated automatic devices (Omron M6) and cuffs of 3 sizes according to arm circumference. Two sets of blood pressure readings were taken separated by 90 min. In each set, blood pressure was measured 3 times at 1–2-min intervals, after resting between 3 and 5 min in a seated position. For the analyses, average systolic and diastolic blood pressure was calculated for those who had at least 3 measurements, after discarding the first reading. HDL-cholesterol was measured with the direct method, by elimination/catalase. Triglycerides were determined by the glycerol phosphate oxidase method [36].

In addition, insulin was determined by immunoradiometric assay. The Homoeostatic Model Assessment for IR Index (HOMA-IR) was calculated by multiplying glucose in mg/dl by insulin in mU/l and dividing by 405 [37]. Finally, high-sensitivity C-reactive protein (hs-CRP) was assessed by latex-enhanced nephelometry to assess chronic inflammation.

##### Assessment of CVD and all-cause mortality

The date and cause of death were ascertained from a computerized search of the vital registry of the Spanish National Institute of Statistics from baseline (2008–2010) to the end of follow-up on December 31, 2017; there is evidence of the completeness, accuracy, and reliability of this vital-status information [38–40]. The date and cause of death was determined from the death certificate by a nosologist and was coded according to the International Classification of Diseases, 10th Revision (ICD-10); specifically, CVD death was encoded as group I00–I99). Censoring was set at the date of death or at the end of the follow-up (December 31, 2017), whichever occurred first.

#### Other variables

At baseline, we collected information on sociodemographic and lifestyle characteristics including age, sex, educational level (no formal or primary education, secondary education, and university) and tobacco smoking (former, never, current) [31]. Also, weight and height were measured twice in each subject under standardized conditions; the BMI was calculated as weight (in kg) divided by squared height (in m) [31]. Finally, study participants reported their main drug treatments and the presence of the following physician-diagnosed diseases: chronic respiratory disease, coronary heart disease, stroke, heart failure, osteoarthritis, cancer, urinary infection, intestinal polyps, stomach ulcers, cirrhosis, Alzheimer’s disease, Parkinson’s disease, and depression requiring treatment.

### Statistical analysis

The association between quartiles of MEDLIFE index and MS, and its individual components, at baseline was summarized with odds ratios (ORs) and their 95% confidence interval (CI), obtained from multivariable logistic regression. In addition, multivariable-adjusted linear regression analyses were built to assess the association between MEDLIFE and HOMA-IR and hs-CRP, with results being summarized with β and 95% CI, where β coefficients represent adjusted differences of means between each of the upper quartiles of MEDLIFE and the lower quartile. P values for linear trend were calculated using quartiles of MEDLIFE as continuous variable. Three multivariable models were built based on the existing evidence that the variables are risk factors for the outcome [41] and observed association with MEDLIFE. Model 1 adjusted for sex, age, and education. Model 2 further adjusted for tobacco smoking (never, former, current), total energy intake (kcal/day), and BMI. And model 3 further adjusted for cancer, respiratory disease, depression, number of morbidities (excluding the previously mentioned conditions), and number of drug treatments. Natural splines adjusted for all covariates as in Model 3 were used to explore the dose–response association between MEDLIFE and biological CVD risk factors as continuous variables (waist circumference, blood glucose, systolic and diastolic blood pressure, triglycerides, HDL-cholesterol, HOMA-IR and hs-CRP).

Cox proportional regression models were fitted to assess the association between quartiles of adherence to the MEDLIFE score at baseline and all-cause mortality and CVD mortality, using the lowest quartile (Q1) as reference. P values for linear trend were calculated using quartiles of MEDLIFE as continuous variable. In addition, hazard ratios (HRs) and 95% CI were calculated for each 2-point increment in the MEDLIFE index. As above, we fitted three hierarchical models and we added a fourth one, which was further adjusted for some intermediate clinical risk factors (components of the MS). The proportional-hazards assumption was tested using the Schoenfeld residuals method. Multivariable-adjusted Kaplan–Meier graphs for survival were also elaborated to represent graphically the association between MEDLIFE and all-cause and CVD mortality.

In ancillary analysis, we stratified by morbidity (presence or not of any chronic condition), sex, age at baseline (or age at death only for the mortality analysis) and BMI. Likelihood ratio tests (with 3 degrees of freedom, the 3 upper quartiles of MEDLIFE and a dichotomous potential effect modifier) were performed to evaluate possible interactions. Other sensitivity analysis included the assessment of the association between each component block of the MEDLIFE and the clinical risk factors and mortality, using restricted cubic spline analysis with 3 knots for all-cause mortality or CVD mortality. Additionally, residual confounding by unmeasured confounders were addressed by calculating the E-value following the methodology described by VaderWeele and Ding [42].

The analyses were performed using Stata version 16.0 (Stata-Corp LLC, College Station, Texas). The survey command was used in the analyses to account for the complex sampling design. All p-values are two-sided and were considered statistically significant at p < 0.05.

## Results

The actual MEDLIFE score ranged between 5 and 23 points in the cohort participants. Their baseline characteristics according to quartiles of the MEDLIFE index are shown in Table 1. Compared to those in the lowest quartile, participants with higher adherence to the MEDLIFE score had lower BMI, number of drug treatments, and morbidities. The frequency of the components of the MS was also lower. Additionally, participants with higher adherence were more likely to have higher education and to be never smokers, and less often had depression or respiratory disease.

**Table 1:** Baseline characteristics of the ENRICA cohort according to quartiles of the MEDLIFE index

	Mediterranean lifestyle index (MEDLIFE)				*p*-value*
	Q1 (5–11 p)	Q2 (12–13 p)	Q3 (14–15 p)	Q4 (16–23 p)	
Number of participants	3042	3435	2917	1696	
MEDLIFE score, range 0–27 p	10.0 (1.16)	12.5 (0.50)	14.5 (0.50)	16.8 (1.09)	< 0.001
Block 1: Mediterranean food consumption, 0–15 p	3.8 (1.25)	5.1 (1.24)	6.2 (1.23)	7.6 (1.38)	< 0.001
Block 2: Mediterranean eating habits, 0–6 p	3.6 (0.87)	3.9 (0.82))	4.2 (0.80)	4.5 (0.77)	< 0.001
Block 3: Mediterranean physical activity, rest, social habits and conviviality, 0–6 p	2.6 (1.13)	3.5 (1.16)	4.1 (1.1)	4.7 (1.0)	< 0.001
Sex, female, %	54.9	53.0	52.5	52.4	0.189
Age, years	47.8 (17.0)	45.8 (16.1)	45.9 (16.0)	46.3 (15.0)	0.311
Educational level, %					< 0.001
≤ Primary	30.3	27.2	24.7	22.6	
Secondary	44.4	43.9	43.5	40.7	
University	25.4	29.0	31.9	36.7	
Smoking status, %					< 0.001
Never	44.2	47.7	50.3	48.5	
Former	21.8	24.3	24.7	29.3	
Current	34.0	28.0	25.0	22.2	
BMI, kg/m^2^	26.8 (4.8)	26.6 (4.6)	26.3 (4.3)	26.2 (4.1)	< 0.001
Energy intake, kcal/day	2274 (944)	2232 (864)	2226 (747)	2238 (683)	0.08
Number of drug treatments	0.71 (1.32)	0.65 (1.26)	0.61 (1.22)	0.62 (1.21)	0.006
Morbidities^a^, %	44.6	40.0	36.3	34.6	< 0.001
Number of morbidities	0.66 (1.0)	0.63 (0.95)	0.56 (0.90)	0.52 (0.88)	< 0.001
Chronic conditions					
Depression, %	7.9	7.2	5.4	4.2	< 0.001
Cancer, %	1.0	0.8	0.9	0.7	0.59
Respiratory disease, %	8.1	7.0	6.5	5.5	0.005
Components of the metabolic syndrome					
Abdominal obesity^b^	36.8	34.4	31.9	28.1	< 0.001
Fasting blood glucose 100–126 mg/dl, n	5.3	5.1	4.8	3.6	0.046
Blood pressure ≥ 130/85 mmHg,	44.9	45.6	42.1	42.9	0.021
Triglycerides ≥ 150 mg/dl	18.7	16.9	15.6	14.2	< 0.001
Low HDL-C^c^	27.9	26.4	23.4	20.5	< 0.001
HOMA-IR	2.1 (1.70)	2.0 (1.6)	1.9 (1.4)	1.8 (1.3)	< 0.001
Hs-CRP, geometric mean (standard error)	0.14 (0.0)	0.14 (0.0)	0.12 (0.0)	0.12 (0.0)	< 0.001

Continuous variables are expressed as mean (standard deviation)

*BMI* body mass index, *kcal* kilocalories, *CVD* cardiovascular disease, *Hs-CRP* highly sensitive C-reactive protein, *HOMA-IR* Homeostatic Model Assessment for Insulin Resistance, *m* meters, *MET* metabolic equivalent of task, *p* points

^*^Continuous variables were compared across categories of MEDLIFE using anova; categorical variables were compared using chi-squared tests

^a^At least one self-reported physician-diagnosed disease (coronary heart disease, stroke and heart failure, osteoarthritis, rheumatoid arthritis, respiratory disease, hip fracture, urinary infection, sleep apnea, depression, Alzheimer’s disease, cirrhosis, Parkinson’s disease, stomach ulcers, intestinal polyps, cataracts, periodontal disease, cancer)

^b^Abdominal obesity: waist circumference ≥ 102 cm in men, and ≥ 88 in women

^c^HDL-C level < 40 mg/dl in men or < 50 in women

**Fig. 1:**
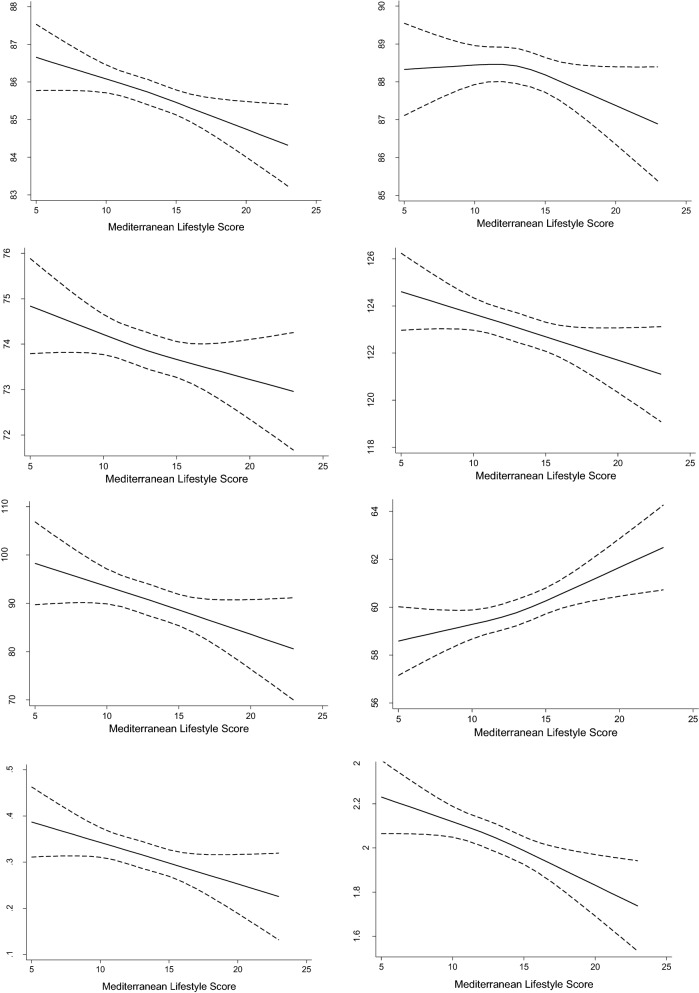
Dose–response association between MEDLIFE, the components of the metabolic syndrome and hs-CRP, HOMA-IR in ENRICA cohort. Analyses were adjusted as in Model 3. *HOMA-IR* Homeostatic Model Assessment for Insulin Resistance, *Hs-CRP* highly sensitive C-reactive protein

A total of 1612 prevalent MS cases were documented. Table 2 shows the OR and 95% CI of the MS, its individual components, and the average values of HOMA-IR and hs-CRP according to quartiles of the MEDLIFE. After multivariable adjustment (model 3) and compared to participants in the lowest quartile of MEDLIFE, the OR for those in the highest quartile was 0.73 (0.58, 0.93) for MS, 0.63 (0.51, 0.80) for abdominal obesity, and 0.76 (0.63, 0.90) for low HDL-cholesterol. In addition, after adjustment for model 2 the inverse association was also statistically significant for elevated triglycerides and high blood pressure (P-trend 0.046). Similarly, higher adherence to the MELDIFE index was associated with lower levels of HOMA-IR and Hs-CRP (P-trend < 0.001). The spline models showed a clear inverse dose–response relationship between the MEDLIFE index and waist circumference, fasting blood glucose, diastolic and systolic blood pressure, triglycerides, HOMA-IR, and Hs-CRP; by contrast, MEDLIFE was directly associated with HDL-cholesterol (Fig. 1).

**Table 2:** Association between quartiles of MEDLIFE index and metabolic syndrome and its components, HOMAR-IR and C-reactive protein in the ENRICA cohort (N = 11,090)

	Q1 (5–11 p)	Q2 (12–13 p)	Q3 (14–15 p)	Q4 (16–23 p)	P-trend
Total number of participants	3042	3435	2917	1696	
Metabolic syndrome, cases	512	530	385	185	
Model 1, OR (95% CI)	1 Ref.	0.91 (0.77, 1.07)	0.80 (0.67, 0.95)	0.66 (0.53, 0.83)	< 0.001
Model 2, OR (95% CI)	1 Ref.	0.88 (0.73, 1.04)	0.86 (0.70, 1.04)	0.71 (0.56, 0.90)	0.007
Model 3, OR (95% CI)	1 Ref.	0.89 (0.74, 1.07)	0.87 (0.72, 1.06)	0.73 (0.58, 0.93)	0.016
Components of the metabolic syndrome					
Abdominal obesity^a^, cases	1111	1176	927	476	
Model 1, OR (95% CI)	1 Ref.	0.91 (0.80, 1.04)	0.82 (0.71, 0.93)	0.68 (0.58, 0.81)	< 0.001
Model 2, OR (95% CI)	1 Ref.	0.82 (0.69, 0.97)	0.83 (0.69, 0.99)	0.63 (0.50, 0.79)	< 0.001
Model 3, OR (95% CI)	1 Ref.	0.82 (0.69, 0.97)	0.84 (0.70, 1.00)	0.63 (0.51, 0.80)	< 0.001
Fasting blood glucose 100–126 mg/dl, cases	161	175	139	60	
Model 1, OR (95% CI)	1 Ref.	1.07 (0.81, 1.41)	1.02 (0.77, 1.36)	0.73 (0.51, 1.06)	0.193
Model 2, OR (95% CI)	1 Ref.	1.04 (0.78, 1.37)	1.04 (0.78, 1.39)	0.74 (0.51, 1.07)	0.247
Model 3, OR (95% CI)	1 Ref.	1.05 (0.79, 1.39)	1.05 (0.79, 1.40)	0.75 (0.52, 1.08)	0.282
Blood pressure ≥ 130/85 mmHg, cases	1354	1555	1217	723	
Model 1, OR (95% CI)	1 Ref.	1.07 (0.94, 1.22)	0.87 (0.75, 1.00)	0.90 (0.76, 1.06)	0.022
Model 2, OR (95% CI)	1 Ref.	1.08 (0.94, 1.25)	0.88 (0.76, 1.01)	0.91 (0.77, 1.08)	*0.046*
Model 3, OR (95% CI)	1 Ref.	1.08 (0.94, 1.24)	0.92 (0.80, 1.05)	0.94 (0.80, 1.10)	0.13
Triglycerides ≥ 150 mg/dl, cases	568	581	455	240	
Model 1, OR (95% CI)	1 Ref.	0.89 (0.77, 1.04)	0.80 (0.67, 0.94)	0.74 (0.61, 0.90)	0.001
Model 2, OR (95% CI)	1 Ref.	0.91 (0.78, 1.06)	0.87 (0.73, 1.03)	0.81 (0.66, 0.99)	0.032
Model 3, OR (95% CI)	1 Ref.	0.92 (0.78, 1.07)	0.88 (0.74, 1.05)	0.83 (0.68, 1.02)	0.053
Low HDL-C^b^, cases	847	905	682	348	
Model 1, OR (95% CI)	1 Ref.	0.87 (0.76, 0.99)	0.80 (0.70, 0.92)	0.68 (0.58, 0.80)	< 0.001
Model 2, OR (95% CI)	1 Ref.	0.89 (0.77, 1.03)	0.86 (0.74, 0.99)	0.75 (0.63, 0.89)	0.001
Model 3, OR (95% CI)	1 Ref.	0.89 (0.78, 1.03)	0.86 (0.74, 0.99)	0.76 (0.63, 0.90)	0.001
HOMA-IR, 95% CI, difference of means					
Model 1, β (95% CI)	0 Ref.	− 0.04 (− 0.12, 0.06)	− 0.11 (− 0.20, − 0.02)	− 0.21 (− 0.31, − 0.11)	< 0.001
Model 2, β (95% CI)	0 Ref.	− 0.03 (− 0.12, 0.05)	− 0.08 (− 0.16, − 0.02)	− 0.18 (− 0.27, − 0.09)	< 0.001
Model 3, β (95% CI)	0 Ref.	− 0.03 (− 0.12, 0.05)	− 0.06 (− 0.15, 0.02)	− 0.17 (− 0.26, − 0.08)	0.001
Hs-CRP (mg/dl)^c^, difference of log-means					
Model 1, β (95% CI)	0 Ref.	0.01 (− 006, 0.08)	− 0.17 (− 0.25, − 0.10)	− 0.13 (− 0.22, − 0.04)	< 0.001
Model 2, β (95% CI)	0 Ref.	0.02 (− 0.05, 0.09)	− 0.14 (− 0.21, − 0.06)	− 0.09 (− 0.17, 0.006)	< 0.001
Model 3, β (95% CI)	0 Ref.	0.03 (− 0.05, 0.09)	− 0.14 (− 0.21, − 0.06)	− 0.09 (− 0.17, 0.02)	0.001

Number of morbidities includes the following self-reported physician-diagnosed diseases (coronary heart disease, stroke and heart failure, osteoarthritis, rheumatoid arthritis, respiratory disease, hip fracture, urinary infection, sleep apnea, depression, Alzheimer’s disease, cirrhosis, Parkinson’s disease, stomach ulcers, intestinal polyps, cataracts, periodontal disease, cancer)

Model 1: adjusted for sex, age, educational level (no formal or primary education, secondary education, university)

Model 2: Adjusted for Model 1 + smoking (never, former, current), total energy intake (kcal/day), and body mass index

Model 3: Model 2 + prevalence of cancer, respiratory disease, depression, number of morbidities (excluding the previously mentioned conditions) and number of drug treatments

*HDL-C* high density lipoprotein cholesterol, *Hs-CRP* highly sensitive C-reactive protein, *HOMA-IR* Homeostatic Model Assessment for Insulin Resistance, *MEDLIFE* Mediterranean lifestyle

^a^Abdominal obesity: waist circumference ≥ 102 cm in men, and ≥ 88 in women

^b^HDL-C level < 40 mg/dl in men or < 50 in women

^c^Values are presented for the logarithmic of the hs-CRP

**Fig. 2:**
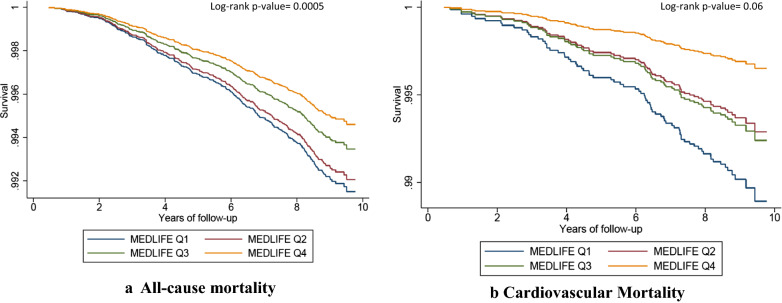
Multivariable-adjusted Kaplan–Meier graph for **a** all cause-death, and **b** cardiovascular death. Adjusted as in Model 4 in Table 3

During a mean follow-up of 8.7 years (SD 0.87), 330 total deaths (including 74 CVD deaths) were ascertained. The cumulative incidence of all-cause mortality is 2% and 0.7% for CVD mortality over a mean follow-up of 8.7 years. The incidence rate of overall mortality is 0.93/100,000 person-year (95% CI 0.84, 1.04); and for CVD deaths is 0.21/100,000 person-year (95% CI 0.17, 0.26). Table 3 shows the multivariable-adjusted HR of all-cause and CVD mortality according to the MEDLIFE index. Compared to participants in the lowest quartile of MEDLIFE score, those in the highest quartile had lower risk of all-cause death [HR 0.58 (95% CI 0.37, 0.90)] and of CVD death [0.31 (0.10, 0.94) (model 3)]. Including the clinical CVD risk factors in the model did not materially change the results for total mortality but turned them into non-significant for CVD death (model 4). Each successive 2-point increase in the MEDLIFE score was significantly associated with a 11–12% reduced relative risk of all-cause death, with a significant linear trend in the models with the highest degree of adjustment. Multivariable-adjusted Kaplan–Meier graphs showed an inverse gradient in all-cause cause and CVD deaths over time according to quartiles of the MEDLIFE index (Fig. 2). Adjusted-restricted cubic splines showed a decrease for both all-cause and CVD mortality (p for linearity = 0.059 for all-cause death and p = 0.196 for CVD death) (Additional file 1: Figure S2).

**Table 3:** Association between quartiles of the MEDLIFE index and risk of all-cause and cardiovascular death over 8.7 years of follow-up

	Categories of adherence to MEDLIFE					p for trend	Per +2-point increment	
	Q1 (4–11 p)	Q2 (12–13 p)		Q3 (14–15 p)	Q4 (16–23 p)			
All-cause mortality								
N	3042	3435		2917	1696			
Deaths	118	106		76	30			
Person-years	9,716,596	10,978,581		9,323,908	5,381,351			
Model 1, HR (95% CI)	1 Ref.	0.92 (0.70, 1.21)		0.68 (0.48, 0.95)	0.54 (0.36, 0.83)	0.004		0.87 (0.79, 0.96)
Model 2, HR (95% CI)	1 Ref.	0.91 (0.69, 1.22)		0.70 (0.49, 0.99)	0.55 (0.36, 0.85)	0.010		0.88 (0.80, 0.97)
Model 3, HR (95% CI)	1 Ref.	0.88 (0.65, 1.18)		0.68 (0.48, 0.96)	0.55 (0.35, 0.85)	0.014		0.88 (0.79, 0.97)
Model 4, HR (95% CI)	1 Ref.	0.92 (0.68, 1.25)		0.72 (0.51, 1.02)	0.58 (0.37, 0.90)	0.025		0.89 (0.81, 0.99)
CVD mortality								
Deaths	29		21	19	5			
Person-years	9,716,596		10,978,581	9,323,908	5,381,351			
Model 1, HR (95% CI)	1 Ref.		0.50 (0.27, 0.93)	0.72 (0.37, 1.37)	0.26 (0.08, 0.79)	0.02		0.78 (0.64, 0.97)
Model 2, HR (95% CI)	1 Ref.		0.55 (0.29, 1.04)	0.82 (0.42, 1.58)	0.30 (0.10, 0.94)	0.06		0.82 (0.67, 1.01)
Model 3, HR (95% CI)	1 Ref.		0.55 (0.28, 1.07)	0.83 (0.43, 1.62)	0.31 (0.10, 0.94)	0.08		0.83 (0.68, 1.02)
Model 4, HR (95% CI)	1 Ref.		0.57 (0.29, 1.14)	0.87 (0.44, 1.70)	0.33 (0.11, 1.02)	0.11		0.84 (0.68, 1.04)

The ENRICA cohort, June 2008 to December 2017

Number of morbidities includes the following self-reported physician-diagnosed diseases (coronary heart disease, stroke and heart failure, osteoarthritis, rheumatoid arthritis, respiratory disease, hip fracture, urinary infection, sleep apnea, depression, Alzheimer’s disease, cirrhosis, Parkinson’s disease, stomach ulcers, intestinal polysp, cataracts, periodontal disease, cancer)

Model 1: adjusted for sex, age, educational level (no formal or primary education, secondary education, university)

Model 2: adjusted for Model 1 + smoking (never, former, current), total energy intake (Kcal/day), and BMI

Model 3: Model 2 + prevalence of cancer, respiratory disease, depression, number of morbidities (excluding the previously mentioned conditions), number of drug treatments

Model 4: Model 3 + other clinical risk factors, including abdominal obesity (waist circumference ≥ 102 cm in men, or ≥ 88 cm in women), fasting blood glucose (100–126 mg/dl and not treated with antidiabetic drugs), high blood pressure (systolic ≥ 130 mmHg or diastolic ≥ 85 mmHg) or receiving antihypertensive drugs, triglycerides ≥ 150 mg/dl and serum high-density lipoprotein (HDL) cholesterol < 40 mg/dl in men or < 50 mg/dl in women)

*HR* hazard ratio, *CI* confidence interval

Results of secondary analyses did not show substantial changes in comparison with the main analysis. In stratified analyses, the association between MEDLIFE and MS did not vary by morbidity, sex, age at baseline, or BMI (All P for interaction > 0.05) (Additional file 1: Table S2**)**. Again, regarding all-cause mortality, no statistically significant interactions were found (Additional file 1: Table S3).

The associations between the individual block components of the MEDLIFE index and MS and mortality were primarily non-significant, except for the Block 3 (physical activity, rest, social habits, and conviviality); specifically, the OR for MS was 0.88 (95% CI 0.83–0.95) while the HR for all-cause mortality was 0.87 (0.78, 0.97) (Additional file 1: Table S4). Within the components of the Block 3, physical activity and collective and non-collective sports were driving the association (Additional file 1: Table S5).

## Discussion

### Main findings

In this cohort, representative of the adult Spanish population, a higher adherence to a Mediterranean lifestyle was associated with lower prevalence of the MS and several biological CVD risk factors as well as lower all-cause and CVD mortality after an average follow-up of almost 9 years. Indeed, we found that higher adherence to the MEDLIFE index was associated with a relatively 45% lower all-cause mortality and 69% relatively lower CVD mortality, but the independent association between MEDLIFE (as continuous) and CVD mortality was absent after adjustment of multiple covariates, maybe due to low number of cases and thus limit of statistical power Physical activity and conviviality was the only block independently associated with the outcomes of the study. Of note, the MEDLIFE index which represents the Mediterranean lifestyle includes food consumption as well as other dietary habits and health behaviors (conviviality, eating in company, rest and social habits) characteristic of the traditional Mediterranean culture and support the importance of cultural habits, beyond mere food habits, as strong determinants of health. This is important because, despite the evidence of the benefits of Mediterranean diet on multiple outcomes [14, 19, 43–45], the fact that our study combines a Mediterranean lifestyle all-inclusive is a clear addition to the literature that emphasizes the synergistic effect of the whole lifestyle rather than their individual components. This message can now be addressed to the population within behavioral counseling, and can possibly translate into improved health outcomes.

Our results add to the evidence that a healthy lifestyle is associated with cardiovascular health and inversely associated with all-cause mortality [21, 22, 26, 46–50]. In the large SUN cohort, comprising university graduates in Spain, a better adherence to a joint healthy lifestyle was associated with lower risk of hypertension [49], MS [51], and CVD [23]. Unlike other scores [22, 23, 25, 26, 48, 49], the MEDLIFE index does not include smoking or BMI because these items (or its absence) do not reflect the Mediterranean lifestyle. Our stratified analyses were robust and the associations were in line with the main analyses by subgroups (by morbidity status, sex, age, age, and BMI) and no significant interaction was found.

### Medlife components and the importance of joint effects

When we assessed the separate effect of the three different blocks of the MEDLIFE, the dimension that captured physical activity, social interaction, rest and conviviality was the only one showing statistically significant results, mainly due to physical activity, collective and non-collective sports. Despite the wide supporting evidence of the benefits of physical activity on cardiovascular health and mortality [52–60], we cannot rule out positive effects of other components such as napping, social interaction, or eating in company that are not usually included in other previously published scores [22, 25, 61]. Our findings may highlight the importance of those components in the context of a healthy Mediterranean dietary pattern. However, we did not assess the isolated effect of each item of the score because we relied on the joint, and possibly synergistic effect of the combination of several behaviors related to the traditional Mediterranean culture; this may contribute to explain why we did not find an association with the other two blocks (capturing food consumption and dietary components). Indeed, lifestyle behaviors are often correlated, thus by simultaneously examining the effect of several lifestyle variables, we accounted for the clustering of healthful types of behavior within the same individual. Additionally, the MEDLIFE score was not designed with the aim of assessing each block separately but to evaluate the comprehensive Mediterranean lifestyle reflecting culture and tradition. Therefore, the main drivers of the association could be different in each population but the overall effect may provide a better understanding of the whole Mediterranean lifestyle, tradition and culture in a holistic approach, and therefore adding the novelty of going beyond the exclusive focus on the foods consumed.

### Lifestyle scores and cardiovascular health

Our results are in line with those of previous studies showing the benefits of a healthy lifestyle on the MS. In a long-term observational study of women, more than one-half of CVD risk factor diagnoses (hypertension, diabetes, high cholesterol) could have been prevented if all of them had optimal levels of 6 lifestyle factors (not smoking, normal BMI, physical activity ≥ 2.5 h/week, television ≤ 7 h/week, diet in top 40% of the Alternative Healthy Eating Index-2010, and 0.1–14.9 g/day of alcohol) [22]. Also a systematic review of 11 interventions found that lifestyle modifications on MS were effective in resolving the proportions of patients with MS in comparison with conventional education [62].

Several studies evaluating other lifestyle scores like the American Heart Association Life Simple 7, that includes 4 lifestyle behaviors (BMI, smoking, diet, physical activity) and 3 clinical risk factors (blood pressure, high cholesterol, and diabetes) have found important inverse associations with CVD and total-mortality in different cohorts [63–68]. In addition, reductions in alcohol intake and energy intake over 1 year in participants with diabetes diagnosis was association with lower hazard of CVD [69]. However, the effect of behavioral factors may be attenuated after the development of the clinical risk factors which are associated with CVD. In our study, after adjustment for the clinical risk factors, the association between MEDLIFE and risk of CVD and all-cause death only slightly changed. While this suggests that the expected protective association of the Mediterranean lifestyle on mortality is substantially greater than that expected only from the reduced frequency of the MS and other clinical risk factors also observed in our study, no etiological conclusion can be drawn.

## Strengths and limitations

Strengths of our study include the long follow-up and large size of the cohort, which is representative of the adult population of a whole country and allows for generalization of the results. Lifestyle information included very detailed questions with a validated dietary history comprising a variety of foods and cooking methods, and a validated physical activity questionnaire. In addition, we used a previously validated lifestyle index (MEDLIFE) reflecting the Mediterranean culture and lifestyle that includes common components across the Mediterranean countries reflecting culture and tradition (social interaction, short nap, meals in company, physical activity with others, etc.). Limitations of this study include potential measurement errors due to the self-reported nature of some of the data, although this bias is most likely to be non-differential and thus would drive the association towards the null. Lifestyle information was only assessed at baseline and, thus, no changes over time were assumed, which could dilute the actual associations. In addition, despite extensive adjustment for known risk factors, some residual confounding may persist; to address this issue we calculated the E-value [42], and it showed that after accounting for confounders measured in this study, the HR of 0.58 for total mortality could only be explained away by an unmeasured confounder that was associated with both the MEDLIFE and mortality with a risk ratio of more than 3.26 (upper CI, 1.12). Thus, given the high E-value (3.26), it seems unlikely that an unmeasured confounder could fully explain away the association between the MEDLIFE and mortality. Additionally, although our results come from a representative Spanish population, generalizability to non-Mediterranean countries or different ethnic groups should be explored. Lastly, although the MEDLIFE aims to be a comprehensive Mediterranean lifestyle index, it is possible that factors not considered, such as spending time in open-air spaces [70], belonging to extended families, or having religious beliefs and practices (mostly Catholic) in Southern Europe may also contribute to cardiovascular health [71, 72].

## Conclusion

In conclusion, a higher adherence to a MEDLIFE index was associated with lower frequency of the MS and reduced all-cause mortality in Spain. Future studies should determine if this also applies to other Mediterranean countries, and whether a Mediterranean-like lifestyle may improve cardiovascular health outside the Mediterranean basin.

## Supplementary Information

**Additional file 1: Table S1.** Description of the Mediterranean Lifestyle (MEDLIFE) index modified for ENRICA. **Table S1.** Description of the Mediterranean Lifestyle (MEDLIFE) index modified for ENRICA. **Table S2.** Subgroup analyses for the association between MEDLIFE quartiles and Metabolic Syndrome. **Table S3.** Subgroup analyses for the association between MEDLIFE quartiles and risk of all-cause death. **Table S4**. Association between each main block of the MEDLIFE index and the metabolic syndrome and all-cause mortality. **Table S5.** Association between each component of Block 3 (Physical Activity and Conviviality) and the metabolic syndrome and all-cause mortality. **Figure S1.** Flowchart of eligible participants in the ENRICA cohort. **Figure S2.** Restricted cubic spline for each 1-point increment of MEDLIFE and risk of all-cause mortality (A) and Cardiovascular Disease (CVD) mortality (B).

## Data Availability

Not applicable.

## Abbreviations

MS: Metabolic syndrome
CVD: Cardiovascular disease
MedDiet: Mediterranean diet
BMI: Body mass index
MEDLIFE: Mediterranean lifestyle index
HR: Hazard ratio
OR: Odds ratio
CI: Confidence interval
HDL-c: High-density lipoprotein cholesterol
HOMA-IR: Homoeostatic Model Assessment for Insulin Resistance Index
hs-CRP: High-sensitivity C-reactive protein
