# Supplementary material for: Association between the Mediterranean lifestyle, metabolic syndrome and mortality: a whole-country cohort in Spain
Source: Cardiovasc Diabetol. 2021 Jan 5;20:5. doi: 10.1186/s12933-020-01195-1 (PMC7786987; doi:10.1186/s12933-020-01195-1)
Supplement: Supplementary file 1 — Additional file 1: Table S1. Description of the Mediterranean Lifestyle (MEDLIFE) index modified for ENRICA. Table S1. Description of the Mediterranean Lifestyle (MEDLIFE) index modified for ENRICA. Table S2. Subgroup analyses for the association between MEDLIFE quartiles and Metabolic Syndrome. Table S3. Subgroup analyses for the association between MEDLIFE quartiles and risk of all-cause death. Table S4. Association between each main block of the MEDLIFE index and the metabolic syndrome and all-cause mortality. Table S5. Association between each component of Block 3 (Physical Activity and Conviviality) and the metabolic syndrome and all-cause mortality. Figure S1. Flowchart of eligible participants in the ENRICA cohort. Figure S2. Restricted cubic spline for each 1-point increment of MEDLIFE and risk of all-cause mortality (A) and Cardiovascular Disease (CVD) mortality (B). [file 12933_2020_1195_MOESM1_ESM.docx]

Additional file 1

**Table S1.** Description of the Mediterranean Lifestyle (MEDLIFE) index modified for ENRICA

| **Score items** | **Components (serving size)** | **Criteria for 1 point**  **in ENRICA** | **% scoring 1 point in ENRICA** |
| --- | --- | --- | --- |
|  | **Block 1: Mediterranean food consumption** | | |
| 1. Sweets | Cookies, chocolate cookies, pastries, donuts, homemade baked goods, store-bought baked goods, muffins (50 g), chocolates (30 g), churros (100 g), turrón and mazapan (35 g) | ≤ 2 servings/wk | 46.0 |
| 2. Red meat | Beef, pork, lamb (100-150 g) | < 2 servings/wk | 60.8 |
| 3. Processed meat | Sausage, soft spicy sausage, bacon (50 g), cured ham (60 g), cooked ham (30 g), hamburger (150 g), liver, organ meats (100-150 g), pâté (25 g) | ≤ 1 serving/wk | 37.9 |
| 4. Eggs | Eggs (1 unit) | 2-4 servings/wk | 34.8 |
| 5. Legumes | Lentils, beans, chickpeas, peas (150 g cooked) | ≥ 2 servings/wk | 37.2 |
| 6. White meat | Chicken/turkey with skin, chicken/turkey without skin, rabbit (100-150 g) | 2 servings/wk | 25.5 |
| 7. Fish/seafood | White fish, fatty fish, codfish, salted or smoked fish, shrimp, octopus, calamari (100-150 g), oysters and shellfish (6 units) | ≥ 2 servings/wk | 73.7 |
| 8. Potatoes | Baked or boiled potatoes (150 g) | ≤ 3 servings/wk | 89.3 |
| 9. Low-fat dairy products | Skim milk, low-fat milk (200 cc), low fat yogurt (125 g), fresh soft cheese (50 g) | 2 servings/d | 6.4 |
| 10. Nuts and olives | Almonds, peanuts, hazelnuts, walnuts (30 g), olives (10 units) | 1-2 servings/d | 10.5 |
| 11. Sofrito | Sauce of olive oil with onion, pepper, other vegetables (250 g) and tomato (150 g) | > 2/4 ingredients above the median | 36.8 |
| 12. Fruit | Orange, banana, apple, pear, kiwi, mango, avocado, peach, apricot, nectarine (1 unit), clementine (2 units), strawberry (6 units), cherries, plums, figs, grapes (1 dessert plate), watermelon, melon (200-250 g), dates and dried fruits (150 g) | 3-6 servings/d | 6.9 |
| 13. Vegetables | Spinach, cauliflower, broccoli, lettuce, carrot, squash, green beans, eggplant, zucchini, cucumber, pepper, asparagus, gazpacho, garden salad, other vegetables (250 g), tomato (150 g) *(excludes potatoes)* | ≥ 2 servings/d | 24.6 |
| 14. Olive oil | Olive oil (1Tbsp=13.5 g) cutoff>36 g | ≥ 3 servings/d | 8.9 |
| 15. Cereals | White bread, whole-grain bread (50g), white or whole grain rice, pasta (150g cooked), pizza dough (200g), breakfast cereal (30g) | 3-6 servings/d | 40.2 |
| **Block 2: Dietary Habits** | | | |
| 16. Wine | Red/white wine (1 glass 100 cc) | women: ≤ 0.5 serving/d  men: ≤ 1 serving/d | 80.2 |
| 18. Limit salt at meals | Do you add salt to foods (eggs, potatoes, fish, fried food)? Do you add salt to vegetables? Do you add salt to salads? | No | 93.3 |
| 19. Preference for whole grain products | Fiber from whole grain cereals | > 6 g/d fiber from cereals | 11.1 |
| 20. Snacks | Potato chips, popcorn or other chips (1s=50g) | < 1 serving/wk | 88.8 |
| 21. Limit snacking between meals | Do you tend to snack in between meals or before going to bed? | NO | 68.5 |
| 22. Limit sugar in beverages (including sugar-sweetened beverages) | Sugar-sweetened beverages + juice (200 cc) | No,< 1/wk, < 1/wk | 57.8 |
| **Block 3 Physical activity, rest and conviviality** | | | |
| 23. Physical activity | Brisk walking, jogging, running, climbing stairs, bicycling, stationary cycling, swimming, dance, aerobic exercise, martial arts, gymnastics, gardening, tennis, soccer, skiing, ice skating, team sports, and other physical activities or sports | > 150 min/wk moderate or 60 min vigorous | 57.3 |
| 24. Nap | Napping throughout the week | >0 & ≤ 30 min/d | 48.5 |
| 25. Hours of sleep | Sleeping throughout the week | 6-8 h/d | 83.7 |
| 26. Watching TV | Watching TV/videos throughout the week | ≤ 2 h/d // <1h | 70.7 |
| 27. Eating in company | Do you have lunch or dinner with friends, family or others? | YES | 56.8 |
| 28. Collective and non-collective sports | Playing soccer, tennis, squash, basketball or other team sports, running, jogging; etc. | ≥ 1 h/wk | 44.6 |

min: minutes, h: hours, d: day, wk: week, cc: cubic centimeter, g: grams, Tbsp: tablespoons, TV: television

**Table S2: Subgroup analyses for the association between MEDLIFE quartiles and Metabolic Syndrome**

|  |  |  | Multivariable adjusted OR (95% CI)^a^ | | | |  |
| --- | --- | --- | --- | --- | --- | --- | --- |
| **Subgroup Analysis** | N | cases | Q1 (4-11 p) | Q2 (12-13 p) | Q3 (14-15 p) | Q4 (16-23 p) | P for interaction |
| **Morbidity ^a^** |  |  |  |  |  |  |  |
| **No** | 6,804 | 817 | 1 Ref. | 1.00 (0.82, 1.21) | **0.79 (0.64, 0.97)** | **0.70 (0.55, 0.90)** | 0.61 |
| **Yes** | 4,286 | 795 | 1 Ref. | 0.87 (0.72, 1.06) | **0.80 (0.64, 0.99)** | **0.59 (0.45, 0.78)** |  |
| **Sex** |  |  |  |  |  |  |  |
| Men | 5,180 | 920 | 1 Ref. | 0.87 (0.72, 1.04) | **0.69 (0.57, 0.83)** | **0.66 (0.52, 0.84)** | 0.32 |
| Women | 5,910 | 692 | 1 Ref. | 1.01 (0.82, 1.24) | 0.93 (0.74, 1.16) | **0.59 (0.44, 0.80)** |  |
| **Age at baseline** |  |  |  |  |  |  |  |
| <55 years old | 7,864 | 892 | 1 Ref. | 0.98 (0.82, 1.17) | **0.80 (0.66, 0.97)** | **0.72 (0.57, 0.92)** | 0.41 |
| ≥55 years old | 3,226 | 720 | 1 Ref. | 0.85 (0.69, 1.04) | **0.75 (0.60, 0.94)** | **0.51 (0.39, 0.69)** |  |
| **BMI** | | | | | | |  |
| <25 kg/m^2^ | 4,388 | 93 | 1 Ref. | 0.75 (0.44, 1.28) | 0.91 (0.53, 1.56) | 0.56 (0.27. 1.17) | 0.56 |
| >=25-kg/m^2^ | 6,702 | 1519 | 1 Ref. | 0.96 (0.83, 1.11) | **0.78 (0.66, 0.91)** | **0.67 (0.55, 0.81)** |  |

BMI: Body Mass Index; OR: Odds ratio, CI: Confidence interval

Model adjusted for sex, age, educational level (no formal or primary education, secondary education, university), smoking (never, former, current), total energy intake (Kcal/day), and BMI, prevalence of cancer, respiratory disease, depression, number of morbidities (excluding the previously mentioned conditions), number of drug treatments. ^a^ At least one self-reported disease (Cardiovascular disease, arthrosis, rheumatoid arthritis, respiratory disease, hip fracture, urinary infection, sleep apnea, depression, Alzheimer, cirrhosis, Parkinson, stomach ulcers, Intestinal polyp, cataracts, periodontal disease, cancer).

**Table S3: Subgroup analyses for the association between MEDLIFE quartiles and risk of all-cause death.**

|  |  | |  | | Multivariable adjusted HR (95% CI)^a^ | | | | | | |  |
| --- | --- | --- | --- | --- | --- | --- | --- | --- | --- | --- | --- | --- |
| **Subgroup Analysis** | N | | cases | | Q1 (4-11 p) | | Q2 (12-13 p) | | Q3 (14-15 p) | Q4 (16-23 p) | | P for interaction |
| **Morbidity** | |  |  |  | |  | |  | | |  |  |
| **No** | | 6,804 | 118 | 1 Ref. | | 1.22 (0.81, 1.83) | | 0.94 (0.60, 1.46) | | | 0.55 (0.29, 1.05) | 0.15 |
| **Yes** | | 4,286 | 212 | 1 Ref. | | **0.75 (0.58, 0.98)** | | **0.74 (0.56, 0.99)** | | | **0.67 (0.46, 0.98)** |  |
| **Sex** | |  |  |  | |  | |  | | |  |  |
| Men | 5,180 | | 195 | | 1 Ref. | | 0.90 (0.63, 1.29) | | 0.70 (0.47, 1.03) | 0.67 (0.40, 1.29) | | 0.88 |
| Women | 5,910 | | 135 | | 1 Ref. | | 0.93 (0.62,1.41) | | 0.80 (0.50, 1.27) | **0.45 (0.22, 0.93)** | |  |
| **Age at baseline** |  | |  | |  | |  | |  |  | |  |
| <55 years old | 7,864 | | 46 | | 1 Ref. | | 0.48 (0.23, 1.01) | | 0.60 (0.28, 1.20) | **0.17 (0.04, 0.73)** | | 0.08 |
| ≥55 years old | 3,226 | | 284 | | 1 Ref. | | 0.81 (0.63, 1.05) | | **0.68 (0.51, 0.91)** | **0.59 (0.40, 0.88)** | |  |
| **Age at death** |  | |  | |  | |  | |  |  | |  |
| <55 years old | 5,936 | | 23 | | 1 Ref. | | 0.52 (0.19, 1.44) | | 0.57 (0.20, 1.61) | 0.18 (0.02, 1.42) | | 0.61 |
| ≥55 years old | 5,154 | | 307 | | 1 Ref. | | 0.85 (0.64, 1.12) | | **0.69 (0.51, 0.93)** | **0.51 (0.34, 0.78)** | |  |
| **BMI** | | | | | | | | | | | |  |
| <25 kg/m^2^ | 6,702 | | 241 | | 1 Ref. | | 0.89 (0.65, 1.21) | | 0.78 (0.56, 1.10) | 0.72 (0.45, 1.15) | | 0.25 |
| ≥25 kg/m^2^ | 4,388 | | 89 | | 1 Ref. | | 1.05 (0.64, 1.74) | | 0.61 (0.34, 1.11) | **0.41 (0.18, 0.95)** | |  |

BMI, Body Mass Index. Model adjusted for sex, age, educational level (no formal or primary education, secondary education, university), smoking (never, former, current), total energy intake (Kcal/day), BMI, prevalence of cancer, respiratory disease, depression, number of morbidities (excluding the previously mentioned conditions), number of drug treatments, and other biological risk factors (high blood pressure, hypertriglyceridemia, high blood glucose, abdominal obesity, low HDL-cholesterol). Number of morbidities includes the following self-reported physician-diagnosed diseases (cardiovascular disease, osteoarthritis, rheumatoid arthritis, respiratory disease, hip fracture, urinary infection, sleep apnea, depression, Alzheimer disease, cirrhosis, Parkinson disease, stomach ulcers, intestinal polyp, cataracts and periodontal disease.

**Table S4**. Association between each main block of the MEDLIFE index and the metabolic syndrome and all-cause mortality

Model adjusted for sex, age, educational level (no formal or primary education, secondary education, university), smoking (never, former, current), total energy intake (Kcal/day), BMI, prevalence of cancer, respiratory disease, depression, number of morbidities (excluding the previous mentioned), number of drug treatment, and other biological risk factors (high blood pressure, hypertriglyceridemia, high blood glucose, abdominal obesity, low HDL-Cholesterol and additionally adjusted for the remaining blocks of the MEDLIFE in each analysis.

**Table S5.** Association between each component of Block 3 (Physical Activity and

Conviviality) and the metabolic syndrome and all-cause mortality

| **Block 3: Physical Activity & conviviality** | **Metabolic syndrome**  **OR (95% CI)** |  | **All-cause mortality**  **HR (95% CI)** |
| --- | --- | --- | --- |
| Physical activity | **0.79 (0.70, 0.89)** |  | **0.63 (0.50, 0.80)** |
| Nap | 0.91 (0.81, 1.03) |  | 1.10 (0.88, 1.38) |
| Hours of sleep | 0.96 (0.82, 1.12) |  | 1.06 (0.81, 1.38) |
| Watching TV | 0.97 (0.85, 1.10) |  | 0.91 (0.72, 1.14) |
| Eating in company | 1.03 (0.92, 1.17) |  | 1.04 (0.82, 1.31) |
| Collective and non-collective sports | **0.70 (0.61, 0.80)** |  | **0.60 (0.45, 0.79)** |

|  | **Metabolic syndrome** | **All-cause mortality** |
| --- | --- | --- |
|  | Odds ratio  (95% CI) | Hazard ratio  (95% CI) |
| Block 1: Food Consumption (0-15 points) | 0.98 (0.93, 1.02) | 0.99 (0.92, 1.07) |
| Block 2: Dietary Habits (0-6 points) | 1.03 (0.93, 1.13) | 1.00 (0.86, 1.18) |
| Block 3: Physical Activity & conviviality (0-6 points) | **0.88 (0.83, 0.95)** | **0.87 (0.78, 0.97)** |

Reference for each category is having 0 points in each item.

Model adjusted for sex, age, educational level (no formal or primary education, secondary education, university), smoking (never, former, current), total energy intake (Kcal/day), BMI, prevalence of cancer, respiratory disease, depression, number of morbidities (excluding the previous mentioned), number of drug treatment, and other biological risk factors (high blood pressure, hypertriglyceridemia, high blood glucose, abdominal obesity, low HDL-Cholesterol and additionally adjusted for the remaining components of the MEDLIFE in each analysis

**n=13,105**

Participants recruited June 2008-October 2010

n=12,184

Participants

**921**

Participants with missing on diet or implausible energy intake

n=12,069

Participants

**115**

Participants with missing on clinical CVD risk factors

**n=** **11,091**

Participants included in analyses

**978**

Participants who had a previous medical diagnosis of CVD (n=262) or diabetes at baseline (n=716)

**Figure S1.** Flowchart of eligible participants in the ENRICA cohort.

1. **All-cause mortality B) CVD mortality**

**Figure S2.** Restricted cubic spline for each 1-point increment of MEDLIFE and risk of all-cause mortality (A) and Cardiovascular Disease (CVD) mortality (B).

Model adjusted for sex, age, educational level (no formal or primary education, secondary education, university), smoking (never, former, current), total energy intake (Kcal/day), BMI, prevalence of cancer, respiratory disease, depression, number of morbidities (excluding the previous mentioned), number of drug treatment, and other biological risk factors (high blood pressure, hypertriglyceridemia, high blood glucose, abdominal obesity, low HDL-Cholesterol
